# Duplicate Vas Deferens Encountered during Inguinal Hernia Repair: A Case Report and Literature Review

**DOI:** 10.1155/2016/8324925

**Published:** 2016-10-20

**Authors:** Maxwell C. Breitinger, Evan H. Roszkowski, Adam J. Bauermeister, Andrew A. Rosenthal

**Affiliations:** ^1^College of Osteopathic Medicine, Nova Southeastern University, 3301 College Ave, Fort Lauderdale, FL 33314, USA; ^2^College of Healthcare Sciences, Nova Southeastern University, 3301 College Ave, Fort Lauderdale, FL 33314, USA; ^3^Department of General Surgery, Cleveland Clinic Florida, 2950 Cleveland Clinic Blvd, Weston, FL 33331, USA; ^4^Memorial Regional Hospital, Division of Acute Care Surgery and Trauma, 3501 Johnson Street, Hollywood, FL 33021, USA

## Abstract

Duplication of the vas deferens is a rare anomaly, defined as the presence of two distinct vasa deferentia within one spermatic cord, with only 28 cases reported worldwide since 1959. We report the case of a 63-year-old man with a duplicate vas deferens, presenting with abdominal pain from bowel obstruction secondary to incarcerated inguinal hernia. Spermatic cord dissection during hernioplasty revealed duplication of the vas deferens within the right spermatic cord. Doppler ultrasonography confirmed absence of waveforms in both vasa deferentia with arterial signal in the accompanying vessel. The hernia was repaired without complication. This report emphasizes recognition of duplicate vas deferens in avoiding iatrogenic injury and optimizing surgical outcome.

## 1. Introduction

Duplication of vas deferens is a congenital anomaly rarely reported in medical literature. It may be encountered during surgery involving the spermatic cord, including inguinal hernia repair, orchiopexy, radical prostatectomy, varicocelectomy, and vasectomy [1]. While the incidence of the anatomic variant has been estimated to be less than 0.05%, only 28 cases (including ours) have been reported worldwide since 1959 [1–25]. Accounting for approximately 50,000 inguinal hernia surgeries performed annually in the United States, with a conservatively estimated anomaly rate of 0.01%, we would expect up to five identified duplicate vas deferens cases per year from hernia repair alone or possibly more when considering other urological surgeries [26]. Therefore, the paucity of information on the condition suggests that either it is rarer than previously estimated or it is highly underrecognized and underreported.

True duplication of the vas deferens, first described in the setting of polyorchidism, refers to a duplicate vas deferens within the spermatic cord [1, 27]. Due to the common embryological origin of the renal collecting system and the ejaculatory system, both of which develop from the mesonephric (Wolffian) duct, true duplication of the vas deferens can be confused with ectopic ureters [27–29]. Failure of the ureteric bud to separate from the mesonephric duct and contact the metanephric blastema to form the renal pelvis and calyces can lead to ectopic ureter connected to the ejaculatory system [27]. This condition has historically held the misnomer double vas deferens, which is associated with ipsilateral renal hypodysplasia or agenesis [7, 27–30]. True duplication, in contrast, has been theorized to result from either a duplication or a transversal division of the central portion of the mesonephric duct during organogenesis [1, 6, 7, 11]. Liang et al. proposed a classification for poly-vasa deferentia, in which the true duplicated vas deferens without polyorchidism is Type I, multiple vas deferens with polyorchidism is Type II, and false poly-vas deferens, ectopic ureter, or double vas deferens is Type III [1]. We have identified our patient as a Type I with true duplication of the vas deferens due to the lack of polyorchidism or renal dysgenesis, confirmed by computerized tomography (CT) [1].

## 2. Case Presentation

A 63-year-old African American male with a history of bilateral inguinal hernia presented with diffuse abdominal pain. This was preceded by several years of aching groin pain, which had increased in severity over the week prior to admission. The patient was single and had no children. On examination, he was found to have a small, reducible left inguinal hernia and a large, incarcerated right inguinal hernia. CT of the abdomen and pelvis demonstrated a right inguinal hernia containing a dilated loop of small bowel with distal decompression, indicating acute obstruction. The kidneys demonstrated symmetrical enhancement bilaterally.

**Figure 1:**
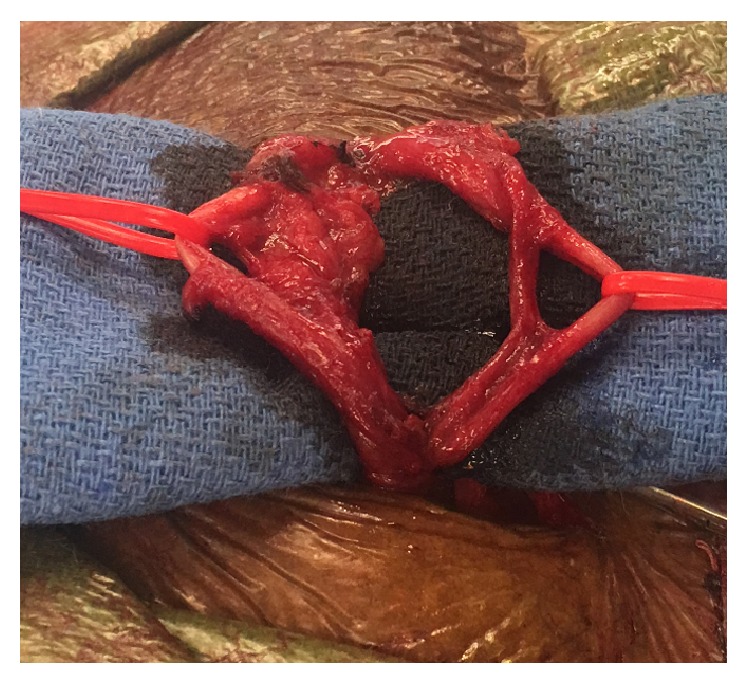
Duplicated vas deferens. 63-year-old male with duplication of vas deferens incidentally discovered during right inguinal hernia repair. The duplicated vasa deferentia have been isolated with vessel loops (photo courtesy of Maxwell C. Breitinger).

The patient underwent an open right inguinal hernia repair under general anesthesia. A significant amount of small bowel was reduced back into the peritoneum after evaluating its viability. A synthetic plug and patch were used to repair the fascial defect. During the dissection of the spermatic cord, it was noted that the patient had two vas deferentia of equal size in the right spermatic cord (Figure 1). Intraoperative audible Doppler Flow Detector was used to establish this abnormal finding; both vas deferentia showed no waveform signal, while a strong arterial signal was detected in the accompanying artery of the vas deferens, which was confirmed to be healthy and viable. The operation was completed without complications and our patient was discharged three days later.

## 3. Discussion

This case involves recognition and preservation of a duplicated vas deferens encountered during open inguinal hernia repair with confirmatory intraoperative Doppler. It is limited by the lack of complete exploration of the distal and proximal courses of the duplicated vas deferentia. We therefore did not determine whether the duplication is partial or complete. In accordance with Kutiyanawala and Johnstone, we did not proceed with dissection in this manner due to lack of contribution to the patient's care [12]. According to Liang et al., identification of a suspected duplicated vas deferens merits urology consultation and tracking of the structure from the internal ring to the epididymis [1]. This practice is strongly recommended if a suspected vas deferens is injured in order to guide prognosis and prevent further complications.

Serious medicolegal complications with regard to fertility can result from failure to recognize and document duplicated vas deferens when encountered [20]. Tolete-Velcek et al. reported a case in which bilateral duplication of the vas deferens was not recognized in a 7-month-old during inguinal hernia repair [20]. Confronted with a pathologic diagnosis of bilateral resection of well-formed segments of vas deferens, the surgeon faced a malpractice suit, permanent entry into a publicly available file, and the uncertainty of the patient's fertility [20]. Prompt reexploration with two consulting surgeons is recommended to ensure intact vasa deferentia bilaterally [20].

Gill et al. argued that aberrant ductal structures in resected pediatric hernia sacs are embryologic remnants, persistent mesonephric tubules which failed to incorporate into the efferent tubules of the testes [31]. These are rarely reported in hernia sacs of adults as they deteriorate by puberty [31]. They concluded that such structures can be distinguished from a true vas deferens by size and histological staining, as they are smaller and surrounded by fibrous tissue with little smooth muscle, in contrast to the muscularis of the vas deferens [31]. However, in cases of true duplication of the vas deferens, a condition which clearly persists into adulthood, size and histology may offer minimal reassurance when a vas deferens is accidentally resected. The potential of surgical trauma to the vas deferens as a cause for infertility was confirmed by Benge and Jordan who demonstrated significant atrophy of the abdominopelvic portion of the vas deferens following ligation or transection in prepubertal humans and rats, affirming that surgical repair of prepubertal vas deferens injury should not be delayed [32].

Duplication of the vas deferens has been implicated in vasectomy failure [21, 33]. Hjarbaek reported a case in which a patient who had undergone an uneventful bilateral vasectomy was readmitted for resterilization due to failure to achieve azoospermia. Reexploration revealed a previously undiscovered duplicate vas deferens, where a portion of which was resected and the ends were ligated, leading to successful sterilization.

Iatrogenic injury to an unrecognized duplicated vas deferens can lead to delayed postoperative complications of spermatic granuloma and chronic pain. Due to the highly antigenic nature of spermatozoa encountered extraluminally from the ductal system, any injury to the vas deferens can lead to extravasation and subsequent development of a nodule surrounding the defect [34]. This development can lead to severe postoperative groin pain and may warrant microsurgical anastomosis [34]. While the more common and clinically significant postoperative complications of hernia recurrence and wound infection should be ruled out first in the setting of postoperative pain and a groin mass, spermatic granuloma should be considered as part of the differential diagnosis [34].

It is important to be aware of the possibility of duplication of the vas deferens, as failure to recognize this condition can lead to postoperative complications including sterilization failure, formation of sperm granuloma with chronic pain, and even reexploration to address fertility concerns. Eight of the 28 cases (29%) were encountered during inguinal hernia repair, highlighting the importance of recognition among not only urologists but also general surgeons [1]. Careful preservation and documentation of duplication of vas deferens upon initial encounter can help prevent legal issues and various postoperative complications.
